# Supplementary material for: Co-exposure risks of pesticides residues and bacterial contamination in fresh fruits and vegetables under smallholder horticultural production systems in Tanzania
Source: PLoS One. 2020 Jul 15;15(7):e0235345. doi: 10.1371/journal.pone.0235345 (PMC7363064; doi:10.1371/journal.pone.0235345)
Supplement: S1 Data — (DOC) [file pone.0235345.s001.doc]

======================================================================

Library Search Report

Data Path : D:\20170731\

Data File : A001100.D

Acq On : 3 Aug 2017 6:23

Operator :

Sample : SAMPLE 0454

Misc :

ALS Vial : 100 Sample Multiplier: 1

Search Libraries: C:\Database\RTLPEST3.L Minimum Quality: 0

Unknown Spectrum: Apex

Integration Events: RTE Integrator - autoint1.e

Pk# RT Area% Library/ID Ref# CAS# Qual

_____________________________________________________________________________

1 7.184 1.51 C:\Database\RTLPEST3.L

No matches found

2 7.430 2.11 C:\Database\RTLPEST3.L

No matches found

3 8.340 3.12 C:\Database\RTLPEST3.L

Cashmeran 585 033704-61-9 9

4 8.826 2.83 C:\Database\RTLPEST3.L

No matches found

5 10.080 2.60 C:\Database\RTLPEST3.L

Binapacryl 403 000485-31-4 4

Dinocap II {CAS # 39300-45-3} 468 999037-03-5 1

Dinocap I 460 039300-45-3 1

6 10.898 1.38 C:\Database\RTLPEST3.L

Endosulfan ether 181 003369-52-6 4

Dinocap IV {CAS # 39300-45-3} 490 999039-03-1 4

Dinocap I 460 039300-45-3 2

7 11.087 41.13 C:\Database\RTLPEST3.L

No matches found

8 11.207 15.12 C:\Database\RTLPEST3.L

Heptachlor 216 000076-44-8 99

Cafenstrole 859 125306-83-4 10

Orbencarb 678 034622-58-7 7

9 12.042 28.50 C:\Database\RTLPEST3.L

No matches found

10 12.254 1.71 C:\Database\RTLPEST3.L

Binapacryl 403 000485-31-4 2

Dinocap I 460 039300-45-3 2

Dinocap III {CAS # 39300-45-3} 481 999038-03-8 2

RESIDUE - ANALYSIS A .M Thu Sep 14 14:26:17 2017 CHEMSTATION

Area Percent Report

Data Path : D:\20170731\

Data File : A001100.D

Acq On : 3 Aug 2017 6:23

Operator :

Sample : SAMPLE 0454

Misc :

ALS Vial : 100 Sample Multiplier: 1

Integration Parameters: rteint.p

Integrator: RTE

Smoothing : ON Filtering: 5

Sampling : 1 Min Area: 3 % of largest Peak

Start Thrs: 0.2 Max Peaks: 100

Stop Thrs : 0 Peak Location: TOP

If leading or trailing edge < 100 prefer < Baseline drop else tangent >

Peak separation: 5

Method : C:\msdchem\1\METHODS\RESIDUE - ANALYSIS A .M

Title : P STD 20 20161011

Signal : TIC: A001100.D\data.ms

peak R.T. first max last PK peak corr. corr. % of

# min scan scan scan TY height area % max. total

--- ----- ----- ---- ---- --- ------- ------- ------ -------

1 7.184 11 16 23 rVB 13991 23130 3.67% 1.511%

2 7.430 52 59 63 rBV2 18316 32287 5.13% 2.109%

3 8.340 211 218 234 rVB 30265 47788 7.59% 3.121%

4 8.826 297 303 314 rBV2 22827 43355 6.89% 2.832%

5 10.080 512 522 532 rVB2 22434 39782 6.32% 2.599%

6 10.898 656 665 677 rBV2 10201 21124 3.35% 1.380%

7 11.087 690 698 713 rBV 317628 629675 100.00% 41.130%

8 11.207 713 719 740 rVB2 120708 231431 36.75% 15.117%

9 12.042 857 865 880 rBV2 150726 436252 69.28% 28.496%

10 12.254 898 902 913 rVB 13403 26117 4.15% 1.706%

Sum of corrected areas: 1530941

RESIDUE - ANALYSIS A .M Thu Sep 14 14:26:49 2017 CHEMSTATION

=======================================================================

Library Search Report

Data Path : D:\20170731\

Data File : 9901099.D

Acq On : 3 Aug 2017 5:42

Operator :

Sample : SAMPLE 0275

Misc :

ALS Vial : 99 Sample Multiplier: 1

Search Libraries: C:\Database\RTLPEST3.L Minimum Quality: 0

Unknown Spectrum: Apex

Integration Events: RTE Integrator - autoint1.e

Pk# RT Area% Library/ID Ref# CAS# Qual

_____________________________________________________________________________

1 7.184 1.62 C:\Database\RTLPEST3.L

No matches found

2 7.430 2.85 C:\Database\RTLPEST3.L

No matches found

3 8.340 4.52 C:\Database\RTLPEST3.L

Cashmeran 585 033704-61-9 5

4 8.826 3.70 C:\Database\RTLPEST3.L

No matches found

5 9.513 2.39 C:\Database\RTLPEST3.L

No matches found

6 10.079 3.53 C:\Database\RTLPEST3.L

Binapacryl 403 000485-31-4 2

7 10.897 2.32 C:\Database\RTLPEST3.L

Endosulfan ether 181 003369-52-6 22

Dinocap II {CAS # 39300-45-3} 468 999037-03-5 4

Dinocap III {CAS # 39300-45-3} 481 999038-03-8 4

8 11.086 42.92 C:\Database\RTLPEST3.L

Dinoseb acetate 233 002813-95-8 1

9 11.206 21.53 C:\Database\RTLPEST3.L

Heptachlor 216 000076-44-8 99

Cafenstrole 859 125306-83-4 7

Phenthoate 332 002597-03-7 4

10 11.836 1.31 C:\Database\RTLPEST3.L

No matches found

11 12.042 8.40 C:\Database\RTLPEST3.L

No matches found

12 12.145 2.99 C:\Database\RTLPEST3.L

No matches found

13 12.254 1.94 C:\Database\RTLPEST3.L

Dinocap III {CAS # 39300-45-3} 481 999038-03-8 9

Binapacryl 403 000485-31-4 4

Dinocap I 460 039300-45-3 2

RESIDUE - ANALYSIS A .M Thu Sep 14 14:25:24 2017 CHEMSTATION

Area Percent Report

Data Path : D:\20170731\

Data File : 9901099.D

Acq On : 3 Aug 2017 5:42

Operator :

Sample : SAMPLE 0275

Misc :

ALS Vial : 99 Sample Multiplier: 1

Integration Parameters: rteint.p

Integrator: RTE

Smoothing : ON Filtering: 5

Sampling : 1 Min Area: 3 % of largest Peak

Start Thrs: 0.2 Max Peaks: 100

Stop Thrs : 0 Peak Location: TOP

If leading or trailing edge < 100 prefer < Baseline drop else tangent >

Peak separation: 5

Method : C:\msdchem\1\METHODS\RESIDUE - ANALYSIS A .M

Title : P STD 20 20161011

Signal : TIC: 9901099.D\data.ms

peak R.T. first max last PK peak corr. corr. % of

# min scan scan scan TY height area % max. total

--- ----- ----- ---- ---- --- ------- ------- ------ -------

1 7.184 12 16 23 rVB 11265 17983 3.77% 1.619%

2 7.430 52 59 63 rBV 18684 31630 6.63% 2.847%

3 8.340 212 218 234 rVB 32493 50257 10.54% 4.524%

4 8.826 297 303 314 rVB2 23154 41098 8.62% 3.699%

5 9.513 417 423 439 rVB 14372 26526 5.56% 2.388%

6 10.079 517 522 532 rVB 23245 39230 8.23% 3.531%

7 10.897 657 665 677 rBV3 12570 25719 5.39% 2.315%

8 11.086 691 698 713 rBV 246942 476807 100.00% 42.919%

9 11.206 713 719 744 rVB2 127302 239192 50.17% 21.530%

10 11.836 824 829 845 rVB3 6854 14504 3.04% 1.306%

11 12.042 858 865 878 rBV2 28316 93279 19.56% 8.396%

12 12.145 880 883 897 rVV3 9999 33168 6.96% 2.986%

13 12.254 898 902 913 rVB 11516 21561 4.52% 1.941%

Sum of corrected areas: 1110954

RESIDUE - ANALYSIS A .M Thu Sep 14 14:25:48 2017 CHEMSTATION

========================================================================

Library Search Report

Data Path : D:\20170731\

Data File : 9801098.D

Acq On : 3 Aug 2017 5:01

Operator :

Sample : SAMPLE 0394

Misc :

ALS Vial : 98 Sample Multiplier: 1

Search Libraries: C:\Database\RTLPEST3.L Minimum Quality: 0

Unknown Spectrum: Apex

Integration Events: RTE Integrator - autoint1.e

Pk# RT Area% Library/ID Ref# CAS# Qual

_____________________________________________________________________________

1 7.327 0.95 C:\Database\RTLPEST3.L

Theobromine 651 000083-67-0 8

TCMTB 357 021564-17-0 2

2 7.373 1.49 C:\Database\RTLPEST3.L

Dinocap IV {CAS # 39300-45-3} 490 999039-03-1 4

Dinocap III {CAS # 39300-45-3} 481 999038-03-8 2

3 7.430 4.13 C:\Database\RTLPEST3.L

Dinocap III {CAS # 39300-45-3} 481 999038-03-8 7

Binapacryl 403 000485-31-4 4

Dinocap I 460 039300-45-3 4

4 7.470 2.44 C:\Database\RTLPEST3.L

No matches found

5 8.014 2.38 C:\Database\RTLPEST3.L

No matches found

6 8.340 5.78 C:\Database\RTLPEST3.L

Cashmeran 585 033704-61-9 5

7 8.523 3.68 C:\Database\RTLPEST3.L

Bendiocarb 96 022781-23-3 12

Cyromazine 135 066215-27-8 1

8 8.826 5.84 C:\Database\RTLPEST3.L

No matches found

9 9.404 0.85 C:\Database\RTLPEST3.L

Fenpropidin 677 067306-00-7 9

10 10.080 4.83 C:\Database\RTLPEST3.L

Binapacryl 403 000485-31-4 4

11 10.440 2.08 C:\Database\RTLPEST3.L

No matches found

12 10.537 0.92 C:\Database\RTLPEST3.L

Dinocap III {CAS # 39300-45-3} 481 999038-03-8 23

Dinocap II {CAS # 39300-45-3} 468 999037-03-5 2

Dinocap I 460 039300-45-3 2

13 10.595 2.08 C:\Database\RTLPEST3.L

Chlordene, trans- 632 003734-48-3 91

Dicyclopentadiene 4 000077-73-6 80

14 10.715 2.88 C:\Database\RTLPEST3.L

No matches found

15 11.081 9.91 C:\Database\RTLPEST3.L

No matches found

16 11.150 2.33 C:\Database\RTLPEST3.L

Pirimiphos-methyl 248 029232-93-7 81

Benzo[g,h,i]perylene 879 000191-24-2 15

Indeno[1,2,3-cd]pyrene 876 000193-39-5 7

17 11.207 27.99 C:\Database\RTLPEST3.L

Heptachlor 216 000076-44-8 99

Cafenstrole 859 125306-83-4 10

Spiroxamine II {CAS # 118134-30-8} 680 999026-03-8 4

18 11.459 0.88 C:\Database\RTLPEST3.L

No matches found

19 11.636 1.06 C:\Database\RTLPEST3.L

No matches found

20 11.836 0.87 C:\Database\RTLPEST3.L

No matches found

21 12.042 9.92 C:\Database\RTLPEST3.L

No matches found

22 12.260 2.47 C:\Database\RTLPEST3.L

Dinocap II {CAS # 39300-45-3} 468 999037-03-5 4

Dinocap III {CAS # 39300-45-3} 481 999038-03-8 3

Binapacryl 403 000485-31-4 2

23 13.032 2.94 C:\Database\RTLPEST3.L

Spiroxamine metabolite (4-tert-but 882 000098-53-3 25

ylcyclohexanone)

Fenpropidin 677 067306-00-7 2

24 13.307 1.30 C:\Database\RTLPEST3.L

No matches found

RESIDUE - ANALYSIS A .M Thu Sep 14 14:24:24 2017 CHEMSTATION

Area Percent Report

Data Path : D:\20170731\

Data File : 9801098.D

Acq On : 3 Aug 2017 5:01

Operator :

Sample : SAMPLE 0394

Misc :

ALS Vial : 98 Sample Multiplier: 1

Integration Parameters: rteint.p

Integrator: RTE

Smoothing : ON Filtering: 5

Sampling : 1 Min Area: 3 % of largest Peak

Start Thrs: 0.2 Max Peaks: 100

Stop Thrs : 0 Peak Location: TOP

If leading or trailing edge < 100 prefer < Baseline drop else tangent >

Peak separation: 5

Method : C:\msdchem\1\METHODS\RESIDUE - ANALYSIS A .M

Title : P STD 20 20161011

Signal : TIC: 9801098.D\data.ms

peak R.T. first max last PK peak corr. corr. % of

# min scan scan scan TY height area % max. total

--- ----- ----- ---- ---- --- ------- ------- ------ -------

1 7.327 31 41 44 rBV4 2840 7914 3.38% 0.946%

2 7.373 44 49 54 rVV 6653 12472 5.33% 1.492%

3 7.430 54 59 63 rVV 18953 34524 14.75% 4.129%

4 7.470 63 66 75 rVB5 11906 20443 8.73% 2.445%

5 8.014 156 161 167 rBV 13170 19923 8.51% 2.383%

6 8.340 212 218 224 rVV 32777 48353 20.66% 5.783%

7 8.523 240 250 267 rBV3 9102 30733 13.13% 3.675%

8 8.826 295 303 320 rVB2 24750 48874 20.88% 5.845%

9 9.404 394 404 408 rBV3 3149 7066 3.02% 0.845%

10 10.080 517 522 530 rBV2 24204 40398 17.26% 4.831%

11 10.440 578 585 590 rBV 8036 17356 7.42% 2.076%

12 10.537 597 602 606 rVV 5122 7718 3.30% 0.923%

13 10.595 606 612 621 rVB2 8494 17352 7.41% 2.075%

14 10.715 623 633 650 rVB3 5902 24106 10.30% 2.883%

15 11.081 691 697 705 rBV2 45065 82860 35.40% 9.909%

16 11.150 705 709 713 rVV 11051 19482 8.32% 2.330%

17 11.207 713 719 741 rVB2 136074 234050 100.00% 27.991%

18 11.459 757 763 774 rVB 4397 7351 3.14% 0.879%

19 11.636 787 794 799 rBV 4616 8890 3.80% 1.063%

20 11.836 825 829 841 rVB7 3107 7256 3.10% 0.868%

21 12.042 858 865 889 rBV2 21594 82964 35.45% 9.922%

22 12.260 897 903 910 rVB 11861 20632 8.82% 2.467%

23 13.032 1030 1038 1058 rBV 8765 24544 10.49% 2.935%

24 13.307 1079 1086 1097 rVB4 4827 10910 4.66% 1.305%

Sum of corrected areas: 836171

RESIDUE - ANALYSIS A .M Thu Sep 14 14:24:49 2017 CHEMSTATION

=========================================================================

Library Search Report

Data Path : D:\20170731\

Data File : 9701097.D

Acq On : 3 Aug 2017 4:21

Operator :

Sample : SAMPLE 0351

Misc :

ALS Vial : 97 Sample Multiplier: 1

Search Libraries: C:\Database\RTLPEST3.L Minimum Quality: 0

Unknown Spectrum: Apex

Integration Events: RTE Integrator - autoint1.e

Pk# RT Area% Library/ID Ref# CAS# Qual

_____________________________________________________________________________

1 7.144 2.78 C:\Database\RTLPEST3.L

Hexestrol 443 000084-16-2 35

Promecarb artifact [5-isopropyl-3- 574 003228-03-3 9

methylphenol]

Thymol 568 000089-83-8 9

2 7.201 2.10 C:\Database\RTLPEST3.L

Azibenzolar-S-methyl 666 135158-54-2 1

3 7.316 1.40 C:\Database\RTLPEST3.L

Theobromine 651 000083-67-0 27

Demephion 59 008065-62-1 9

TCMTB 357 021564-17-0 2

4 7.373 0.66 C:\Database\RTLPEST3.L

Dinocap III {CAS # 39300-45-3} 481 999038-03-8 10

5 7.430 1.22 C:\Database\RTLPEST3.L

Dinocap IV {CAS # 39300-45-3} 490 999039-03-1 9

Dinocap III {CAS # 39300-45-3} 481 999038-03-8 4

Dinocap I 460 039300-45-3 2

6 7.476 0.88 C:\Database\RTLPEST3.L

Dinocap II {CAS # 39300-45-3} 468 999037-03-5 9

Dinocap IV {CAS # 39300-45-3} 490 999039-03-1 9

Dinocap I 460 039300-45-3 7

7 7.774 2.75 C:\Database\RTLPEST3.L

Propamocarb 32 024579-73-5 9

Vamidothion 353 002275-23-2 4

8 7.922 1.17 C:\Database\RTLPEST3.L

Ethiofencarb 188 029973-13-5 2

9 8.014 1.17 C:\Database\RTLPEST3.L

No matches found

10 8.231 0.56 C:\Database\RTLPEST3.L

No matches found

11 8.277 2.01 C:\Database\RTLPEST3.L

Propamocarb 32 024579-73-5 2

12 8.340 1.77 C:\Database\RTLPEST3.L

Cashmeran 585 033704-61-9 8

13 8.523 2.58 C:\Database\RTLPEST3.L

Cyromazine 135 066215-27-8 38

14 8.655 0.78 C:\Database\RTLPEST3.L

Dinocap III {CAS # 39300-45-3} 481 999038-03-8 12

Dinocap IV {CAS # 39300-45-3} 490 999039-03-1 2

Dinocap I 460 039300-45-3 2

15 8.752 5.62 C:\Database\RTLPEST3.L

No matches found

16 8.826 1.59 C:\Database\RTLPEST3.L

Dinocap II {CAS # 39300-45-3} 468 999037-03-5 1

17 9.050 0.52 C:\Database\RTLPEST3.L

No matches found

18 9.175 1.86 C:\Database\RTLPEST3.L

Propamocarb 32 024579-73-5 2

19 9.267 0.67 C:\Database\RTLPEST3.L

Dimethoate 118 000060-51-5 6

20 9.399 1.02 C:\Database\RTLPEST3.L

No matches found

21 9.622 2.26 C:\Database\RTLPEST3.L

N-1-Naphthylacetamide 193 000575-36-0 9

22 9.977 0.99 C:\Database\RTLPEST3.L

Carbofuran-7-phenol 572 001563-38-8 50

Diisobutyl phthalate 652 000084-69-5 9

Carbofuran 125 001563-66-2 9

23 10.080 1.41 C:\Database\RTLPEST3.L

Binapacryl 403 000485-31-4 9

24 10.142 0.85 C:\Database\RTLPEST3.L

Molinate 57 002212-67-1 4

25 10.205 0.54 C:\Database\RTLPEST3.L

No matches found

26 10.331 0.57 C:\Database\RTLPEST3.L

No matches found

27 10.394 1.04 C:\Database\RTLPEST3.L

Diethyl phthalate 72 000084-66-2 38

Diamyl phthalate 364 000131-18-0 9

Di-n-butylphthalate 254 000084-74-2 9

28 10.440 1.12 C:\Database\RTLPEST3.L

No matches found

29 10.480 2.89 C:\Database\RTLPEST3.L

Dinocap III {CAS # 39300-45-3} 481 999038-03-8 9

Dinocap I 460 039300-45-3 2

30 10.537 1.26 C:\Database\RTLPEST3.L

Dinocap III {CAS # 39300-45-3} 481 999038-03-8 35

Dinocap I 460 039300-45-3 2

Dinocap IV {CAS # 39300-45-3} 490 999039-03-1 1

31 10.600 1.37 C:\Database\RTLPEST3.L

Chlordene, trans- 632 003734-48-3 62

Dicyclopentadiene 4 000077-73-6 39

32 10.709 1.94 C:\Database\RTLPEST3.L

No matches found

33 10.755 0.75 C:\Database\RTLPEST3.L

No matches found

34 10.892 1.16 C:\Database\RTLPEST3.L

Demephion 59 008065-62-1 2

35 11.087 16.08 C:\Database\RTLPEST3.L

No matches found

36 11.207 13.46 C:\Database\RTLPEST3.L

Heptachlor 216 000076-44-8 99

Cafenstrole 859 125306-83-4 7

Spiroxamine II {CAS # 118134-30-8} 680 999026-03-8 4

37 11.624 4.30 C:\Database\RTLPEST3.L

MCPB methyl ester 154 057153-18-1 7

38 11.842 0.51 C:\Database\RTLPEST3.L

Exaltolide [15-Pentadecanolide] 638 000106-02-5 14

39 12.048 7.78 C:\Database\RTLPEST3.L

No matches found

40 12.254 1.11 C:\Database\RTLPEST3.L

Dinocap I 460 039300-45-3 2

Dinocap II {CAS # 39300-45-3} 468 999037-03-5 2

Binapacryl 403 000485-31-4 2

41 13.169 0.58 C:\Database\RTLPEST3.L

No matches found

42 13.301 1.23 C:\Database\RTLPEST3.L

2-ethyl-6-methylaniline 564 024549-06-2 9

4-Isopropylaniline 562 000099-88-7 7

43 13.965 0.78 C:\Database\RTLPEST3.L

No matches found

44 14.114 1.40 C:\Database\RTLPEST3.L

Traseolide 646 068140-48-7 9

Barban 381 000101-27-9 6

4-Chlorophenyl isocyanate 558 000104-12-1 2

45 14.743 0.52 C:\Database\RTLPEST3.L

Quinoclamine 244 002797-51-5 4

46 15.698 1.01 C:\Database\RTLPEST3.L

No matches found

RESIDUE - ANALYSIS A .M Thu Sep 14 14:23:21 2017 CHEMSTATION

Area Percent Report

Data Path : D:\20170731\

Data File : 9701097.D

Acq On : 3 Aug 2017 4:21

Operator :

Sample : SAMPLE 0351

Misc :

ALS Vial : 97 Sample Multiplier: 1

Integration Parameters: rteint.p

Integrator: RTE

Smoothing : ON Filtering: 5

Sampling : 1 Min Area: 3 % of largest Peak

Start Thrs: 0.2 Max Peaks: 100

Stop Thrs : 0 Peak Location: TOP

If leading or trailing edge < 100 prefer < Baseline drop else tangent >

Peak separation: 5

Method : C:\msdchem\1\METHODS\RESIDUE - ANALYSIS A .M

Title : P STD 20 20161011

Signal : TIC: 9701097.D\data.ms

peak R.T. first max last PK peak corr. corr. % of

# min scan scan scan TY height area % max. total

--- ----- ----- ---- ---- --- ------- ------- ------ -------

1 7.144 3 9 14 rVB 52417 84683 17.28% 2.778%

2 7.201 14 19 31 rVB 34492 63880 13.03% 2.096%

3 7.316 31 39 45 rBV2 16117 42750 8.72% 1.402%

4 7.373 45 49 54 rVB 13320 20044 4.09% 0.658%

5 7.430 54 59 63 rBV 20026 37155 7.58% 1.219%

6 7.476 64 67 73 rVV4 14233 26847 5.48% 0.881%

7 7.774 107 119 127 rBV3 46113 83737 17.09% 2.747%

8 7.922 139 145 150 rVB 21953 35740 7.29% 1.173%

9 8.014 156 161 167 rVB 24456 35717 7.29% 1.172%

10 8.231 190 199 202 rBV3 7675 16934 3.46% 0.556%

11 8.277 202 207 213 rVB2 35135 61142 12.48% 2.006%

12 8.340 213 218 223 rVB 37225 53988 11.02% 1.771%

13 8.523 241 250 254 rBV 41088 78652 16.05% 2.580%

14 8.655 267 273 283 rVB4 10179 23830 4.86% 0.782%

15 8.752 283 290 297 rBV 110178 171422 34.98% 5.624%

16 8.826 297 303 317 rVB3 24895 48442 9.88% 1.589%

17 9.050 338 342 348 rVB4 8516 15743 3.21% 0.516%

18 9.175 359 364 371 rVB 36142 56748 11.58% 1.862%

19 9.267 371 380 387 rBV8 6986 20416 4.17% 0.670%

20 9.399 396 403 411 rVB2 15671 31065 6.34% 1.019%

21 9.622 435 442 448 rBV2 34453 68930 14.06% 2.261%

22 9.977 498 504 510 rBV3 16652 30293 6.18% 0.994%

23 10.080 516 522 528 rVB2 26825 42931 8.76% 1.408%

24 10.142 528 533 539 rVV 15550 25867 5.28% 0.849%

25 10.205 539 544 556 rVB4 7951 16554 3.38% 0.543%

26 10.331 557 566 571 rVV7 5994 17362 3.54% 0.570%

27 10.394 571 577 581 rVV3 17011 31756 6.48% 1.042%

28 10.440 581 585 588 rVV 18165 33996 6.94% 1.115%

29 10.480 588 592 597 rVV 50280 88205 18.00% 2.894%

30 10.537 598 602 607 rVV 19804 38266 7.81% 1.255%

31 10.600 607 613 622 rVV3 14876 41727 8.51% 1.369%

32 10.709 625 632 637 rVV2 27221 58990 12.04% 1.935%

33 10.755 637 640 649 rVB3 14204 22728 4.64% 0.746%

34 10.892 655 664 670 rBV3 17708 35432 7.23% 1.162%

35 11.087 690 698 706 rBV 281323 490115 100.00% 16.079%

36 11.207 713 719 737 rVB3 232122 410395 83.73% 13.464%

37 11.624 785 792 800 rBV 63583 130967 26.72% 4.297%

38 11.842 825 830 834 rBV4 8003 15583 3.18% 0.511%

39 12.048 859 866 879 rBV3 69899 237203 48.40% 7.782%

40 12.254 898 902 920 rVB 15241 33811 6.90% 1.109%

41 13.169 1056 1062 1070 rVB 10107 17764 3.62% 0.583%

42 13.301 1076 1085 1098 rVB7 10875 37590 7.67% 1.233%

43 13.965 1193 1201 1209 rBV2 10629 23687 4.83% 0.777%

44 14.114 1218 1227 1246 rVB 13979 42538 8.68% 1.396%

45 14.743 1329 1337 1362 rVB3 4155 15779 3.22% 0.518%

46 15.699 1490 1504 1517 rBV2 8635 30767 6.28% 1.009%

Sum of corrected areas: 3048171

RESIDUE - ANALYSIS A .M Thu Sep 14 14:23:47 2017 CHEMSTATION

=================================================================

Library Search Report

Data Path : D:\20170731\

Data File : 9601096.D

Acq On : 3 Aug 2017 3:40

Operator :

Sample : SAMPLE 0475

Misc :

ALS Vial : 96 Sample Multiplier: 1

Search Libraries: C:\Database\RTLPEST3.L Minimum Quality: 0

Unknown Spectrum: Apex

Integration Events: RTE Integrator - autoint1.e

Pk# RT Area% Library/ID Ref# CAS# Qual

_____________________________________________________________________________

1 7.138 1.06 C:\Database\RTLPEST3.L

Propargite metabolite [Cyclohexano 672 999004-03-4 12

l, 2-(4-tert-butylphenoxy)]

Promecarb 103 002631-37-0 10

Hexestrol 443 000084-16-2 10

2 7.184 1.81 C:\Database\RTLPEST3.L

No matches found

3 7.430 3.71 C:\Database\RTLPEST3.L

Dinocap II {CAS # 39300-45-3} 468 999037-03-5 2

Binapacryl 403 000485-31-4 2

4 8.340 5.23 C:\Database\RTLPEST3.L

Cashmeran 585 033704-61-9 5

5 8.826 4.64 C:\Database\RTLPEST3.L

No matches found

6 10.080 4.54 C:\Database\RTLPEST3.L

Binapacryl 403 000485-31-4 2

7 10.892 1.61 C:\Database\RTLPEST3.L

Dinocap III {CAS # 39300-45-3} 481 999038-03-8 9

Dinocap I 460 039300-45-3 8

Demephion 59 008065-62-1 4

8 11.087 33.87 C:\Database\RTLPEST3.L

No matches found

9 11.207 21.99 C:\Database\RTLPEST3.L

Heptachlor 216 000076-44-8 99

Cafenstrole 859 125306-83-4 9

Spiroxamine II {CAS # 118134-30-8} 680 999026-03-8 4

10 11.836 1.12 C:\Database\RTLPEST3.L

No matches found

11 12.042 15.64 C:\Database\RTLPEST3.L

No matches found

12 12.254 3.64 C:\Database\RTLPEST3.L

Dinocap I 460 039300-45-3 9

Dinocap II {CAS # 39300-45-3} 468 999037-03-5 2

Binapacryl 403 000485-31-4 1

13 13.307 1.13 C:\Database\RTLPEST3.L

Quinoclamine 244 002797-51-5 4

RESIDUE - ANALYSIS A .M Thu Sep 14 14:22:27 2017 CHEMSTATION

Area Percent Report

Data Path : D:\20170731\

Data File : 9601096.D

Acq On : 3 Aug 2017 3:40

Operator :

Sample : SAMPLE 0475

Misc :

ALS Vial : 96 Sample Multiplier: 1

Integration Parameters: rteint.p

Integrator: RTE

Smoothing : ON Filtering: 5

Sampling : 1 Min Area: 3 % of largest Peak

Start Thrs: 0.2 Max Peaks: 100

Stop Thrs : 0 Peak Location: TOP

If leading or trailing edge < 100 prefer < Baseline drop else tangent >

Peak separation: 5

Method : C:\msdchem\1\METHODS\RESIDUE - ANALYSIS A .M

Title : P STD 20 20161011

Signal : TIC: 9601096.D\data.ms

peak R.T. first max last PK peak corr. corr. % of

# min scan scan scan TY height area % max. total

--- ----- ----- ---- ---- --- ------- ------- ------ -------

1 7.138 3 8 12 rBV 5787 10241 3.13% 1.061%

2 7.184 12 16 23 rVB 11216 17485 5.35% 1.811%

3 7.430 51 59 63 rBV2 19889 35853 10.97% 3.714%

4 8.340 212 218 234 rVB 32892 50494 15.44% 5.231%

5 8.826 297 303 314 rBV2 23595 44819 13.71% 4.643%

6 10.080 510 522 532 rBV2 23803 43836 13.41% 4.541%

7 10.892 656 664 677 rBV2 6835 15529 4.75% 1.609%

8 11.087 690 698 706 rBV 181858 326931 100.00% 33.868%

9 11.207 713 719 742 rVB2 120427 212304 64.94% 21.993%

10 11.836 824 829 844 rBV5 4035 10796 3.30% 1.118%

11 12.042 858 865 879 rBV3 44874 150973 46.18% 15.640%

12 12.254 898 902 933 rVB 14003 35134 10.75% 3.640%

13 13.307 1080 1086 1101 rVB2 4693 10914 3.34% 1.131%

Sum of corrected areas: 965309

RESIDUE - ANALYSIS A .M Thu Sep 14 14:22:53 2017 CHEMSTATION

========================================================================

Library Search Report

Data Path : D:\20170731\

Data File : 9501095.D

Acq On : 3 Aug 2017 2:59

Operator :

Sample : SAMPLE 0431

Misc :

ALS Vial : 95 Sample Multiplier: 1

Search Libraries: C:\Database\RTLPEST3.L Minimum Quality: 0

Unknown Spectrum: Apex

Integration Events: RTE Integrator - autoint1.e

Pk# RT Area% Library/ID Ref# CAS# Qual

_____________________________________________________________________________

1 7.430 1.77 C:\Database\RTLPEST3.L

Dinocap II {CAS # 39300-45-3} 468 999037-03-5 1

2 7.859 1.72 C:\Database\RTLPEST3.L

No matches found

3 8.340 3.34 C:\Database\RTLPEST3.L

Cashmeran 585 033704-61-9 9

4 8.826 2.53 C:\Database\RTLPEST3.L

No matches found

5 9.730 2.88 C:\Database\RTLPEST3.L

No matches found

6 10.079 2.21 C:\Database\RTLPEST3.L

Binapacryl 403 000485-31-4 4

Dinocap I 460 039300-45-3 1

7 11.087 34.20 C:\Database\RTLPEST3.L

Dinoseb acetate 233 002813-95-8 1

8 11.207 11.52 C:\Database\RTLPEST3.L

Heptachlor 216 000076-44-8 99

Cafenstrole 859 125306-83-4 10

Ethiolate 15 002941-55-1 4

9 12.042 23.64 C:\Database\RTLPEST3.L

No matches found

10 12.145 12.22 C:\Database\RTLPEST3.L

No matches found

11 12.254 3.97 C:\Database\RTLPEST3.L

No matches found

RESIDUE - ANALYSIS A .M Thu Sep 14 14:21:36 2017 CHEMSTATION

Area Percent Report

Data Path : D:\20170731\

Data File : 9501095.D

Acq On : 3 Aug 2017 2:59

Operator :

Sample : SAMPLE 0431

Misc :

ALS Vial : 95 Sample Multiplier: 1

Integration Parameters: rteint.p

Integrator: RTE

Smoothing : ON Filtering: 5

Sampling : 1 Min Area: 3 % of largest Peak

Start Thrs: 0.2 Max Peaks: 100

Stop Thrs : 0 Peak Location: TOP

If leading or trailing edge < 100 prefer < Baseline drop else tangent >

Peak separation: 5

Method : C:\msdchem\1\METHODS\RESIDUE - ANALYSIS A .M

Title : P STD 20 20161011

Signal : TIC: 9501095.D\data.ms

peak R.T. first max last PK peak corr. corr. % of

# min scan scan scan TY height area % max. total

--- ----- ----- ---- ---- --- ------- ------- ------ -------

1 7.430 53 59 63 rBV 20095 33275 5.16% 1.765%

2 7.859 128 134 141 rBV 20011 32388 5.02% 1.718%

3 8.340 208 218 237 rVB 34685 62865 9.75% 3.335%

4 8.826 291 303 321 rBV2 23826 47733 7.40% 2.532%

5 9.730 454 461 467 rBV 35995 54340 8.43% 2.883%

6 10.079 510 522 532 rBV 22606 41712 6.47% 2.213%

7 11.087 691 698 713 rBV 331881 644741 100.00% 34.204%

8 11.207 713 719 730 rVB2 119961 217102 33.67% 11.518%

9 12.042 857 865 878 rBV2 157448 445692 69.13% 23.645%

10 12.145 879 883 897 rVV2 82600 230248 35.71% 12.215%

11 12.254 899 902 932 rVB3 22682 74864 11.61% 3.972%

Sum of corrected areas: 1884960

RESIDUE - ANALYSIS A .M Thu Sep 14 14:21:58 2017 CHEMSTATION

===================================================================

Library Search Report

Data Path : D:\20170731\

Data File : 9401094.D

Acq On : 3 Aug 2017 2:18

Operator :

Sample : SAMPLE 0283

Misc :

ALS Vial : 94 Sample Multiplier: 1

Search Libraries: C:\Database\RTLPEST3.L Minimum Quality: 0

Unknown Spectrum: Apex

Integration Events: RTE Integrator - autoint1.e

Pk# RT Area% Library/ID Ref# CAS# Qual

_____________________________________________________________________________

1 7.201 3.57 C:\Database\RTLPEST3.L

Azibenzolar-S-methyl 666 135158-54-2 1

2 7.316 1.04 C:\Database\RTLPEST3.L

Theobromine 651 000083-67-0 9

Demephion 59 008065-62-1 2

TCMTB 357 021564-17-0 2

3 7.373 1.32 C:\Database\RTLPEST3.L

Dinocap III {CAS # 39300-45-3} 481 999038-03-8 9

4 7.430 2.41 C:\Database\RTLPEST3.L

No matches found

5 7.476 2.43 C:\Database\RTLPEST3.L

Dinocap III {CAS # 39300-45-3} 481 999038-03-8 9

Dinocap II {CAS # 39300-45-3} 468 999037-03-5 4

Dinocap I 460 039300-45-3 4

6 8.014 1.10 C:\Database\RTLPEST3.L

No matches found

7 8.340 3.48 C:\Database\RTLPEST3.L

Cashmeran 585 033704-61-9 10

8 8.523 1.39 C:\Database\RTLPEST3.L

Cyromazine 135 066215-27-8 42

Bendiocarb 96 022781-23-3 25

9 8.826 3.26 C:\Database\RTLPEST3.L

No matches found

10 9.484 1.40 C:\Database\RTLPEST3.L

Dinocap III {CAS # 39300-45-3} 481 999038-03-8 22

Dinocap II {CAS # 39300-45-3} 468 999037-03-5 2

Dinocap IV {CAS # 39300-45-3} 490 999039-03-1 2

11 9.622 0.77 C:\Database\RTLPEST3.L

No matches found

12 10.079 2.80 C:\Database\RTLPEST3.L

Binapacryl 403 000485-31-4 4

Dinocap II {CAS # 39300-45-3} 468 999037-03-5 4

13 10.383 0.77 C:\Database\RTLPEST3.L

Diamyl phthalate 364 000131-18-0 35

Diisobutyl phthalate 652 000084-69-5 25

Ethofumesate, 2-Keto 660 026244-33-7 17

14 10.480 6.44 C:\Database\RTLPEST3.L

Dinocap I 460 039300-45-3 2

Dinocap IV {CAS # 39300-45-3} 490 999039-03-1 1

15 10.537 2.40 C:\Database\RTLPEST3.L

Dinocap III {CAS # 39300-45-3} 481 999038-03-8 4

Dinocap II {CAS # 39300-45-3} 468 999037-03-5 2

Dinocap I 460 039300-45-3 2

16 10.612 3.05 C:\Database\RTLPEST3.L

Dinocap III {CAS # 39300-45-3} 481 999038-03-8 32

Dinocap II {CAS # 39300-45-3} 468 999037-03-5 2

17 10.715 2.52 C:\Database\RTLPEST3.L

No matches found

18 10.892 0.57 C:\Database\RTLPEST3.L

Vamidothion 353 002275-23-2 2

Demephion 59 008065-62-1 1

19 11.081 11.84 C:\Database\RTLPEST3.L

No matches found

20 11.149 1.83 C:\Database\RTLPEST3.L

Pirimiphos-methyl 248 029232-93-7 35

Benzo[g,h,i]perylene 879 000191-24-2 9

Indeno[1,2,3-cd]pyrene 876 000193-39-5 7

21 11.207 17.64 C:\Database\RTLPEST3.L

Heptachlor 216 000076-44-8 99

Cafenstrole 859 125306-83-4 9

Spiroxamine I 663 118134-30-8 4

22 11.624 1.87 C:\Database\RTLPEST3.L

No matches found

23 11.842 0.73 C:\Database\RTLPEST3.L

No matches found

24 11.979 0.88 C:\Database\RTLPEST3.L

Endosulfan ether 181 003369-52-6 43

Dinocap III {CAS # 39300-45-3} 481 999038-03-8 38

Dinocap II {CAS # 39300-45-3} 468 999037-03-5 37

25 12.048 17.47 C:\Database\RTLPEST3.L

No matches found

26 12.259 2.32 C:\Database\RTLPEST3.L

Toxaphene Parlar 50 797 066860-80-8 9

Binapacryl 403 000485-31-4 4

Dinocap III {CAS # 39300-45-3} 481 999038-03-8 4

27 13.032 2.67 C:\Database\RTLPEST3.L

Spiroxamine metabolite (4-tert-but 882 000098-53-3 35

ylcyclohexanone)

Fenpropidin 677 067306-00-7 2

28 13.307 0.81 C:\Database\RTLPEST3.L

No matches found

29 13.959 0.65 C:\Database\RTLPEST3.L

Pyridate 542 055512-33-9 4

30 14.033 0.57 C:\Database\RTLPEST3.L

No matches found

RESIDUE - ANALYSIS A .M Thu Sep 14 14:20:37 2017 CHEMSTATION

Area Percent Report

Data Path : D:\20170731\

Data File : 9401094.D

Acq On : 3 Aug 2017 2:18

Operator :

Sample : SAMPLE 0283

Misc :

ALS Vial : 94 Sample Multiplier: 1

Integration Parameters: rteint.p

Integrator: RTE

Smoothing : ON Filtering: 5

Sampling : 1 Min Area: 3 % of largest Peak

Start Thrs: 0.2 Max Peaks: 100

Stop Thrs : 0 Peak Location: TOP

If leading or trailing edge < 100 prefer < Baseline drop else tangent >

Peak separation: 5

Method : C:\msdchem\1\METHODS\RESIDUE - ANALYSIS A .M

Title : P STD 20 20161011

Signal : TIC: 9401094.D\data.ms

peak R.T. first max last PK peak corr. corr. % of

# min scan scan scan TY height area % max. total

--- ----- ----- ---- ---- --- ------- ------- ------ -------

1 7.201 13 19 31 rVB 28338 50545 20.22% 3.568%

2 7.316 31 39 44 rBV2 5928 14701 5.88% 1.038%

3 7.373 44 49 54 rVV 11734 18770 7.51% 1.325%

4 7.430 54 59 63 rVV 18713 34160 13.67% 2.411%

5 7.476 63 67 76 rVB4 15728 34378 13.76% 2.426%

6 8.014 151 161 167 rBV 9455 15629 6.25% 1.103%

7 8.340 212 218 224 rVV 33335 49310 19.73% 3.480%

8 8.523 242 250 261 rBV3 6820 19668 7.87% 1.388%

9 8.826 297 303 319 rVB2 24318 46117 18.45% 3.255%

10 9.484 408 418 432 rVB2 8889 19778 7.91% 1.396%

11 9.622 433 442 448 rBV2 4697 10947 4.38% 0.773%

12 10.079 516 522 530 rBV2 23436 39690 15.88% 2.801%

13 10.383 568 575 580 rBV3 4786 10894 4.36% 0.769%

14 10.480 580 592 597 rVV 46532 91216 36.50% 6.438%

15 10.537 598 602 607 rVV 17625 34028 13.62% 2.402%

16 10.612 607 615 623 rVV2 19220 43236 17.30% 3.052%

17 10.715 623 633 651 rVB3 8917 35707 14.29% 2.520%

18 10.892 658 664 669 rBV 5027 8046 3.22% 0.568%

19 11.081 691 697 705 rBV 92572 167731 67.11% 11.839%

20 11.149 706 709 713 rVV 15329 25959 10.39% 1.832%

21 11.207 713 719 739 rVB2 143956 249921 100.00% 17.640%

22 11.624 786 792 800 rBV 12506 26453 10.58% 1.867%

23 11.842 824 830 839 rBV5 4894 10387 4.16% 0.733%

24 11.979 844 854 858 rVV2 5692 12536 5.02% 0.885%

25 12.048 858 866 897 rVV2 71610 247482 99.02% 17.468%

26 12.259 898 903 925 rVB 13879 32907 13.17% 2.323%

27 13.032 1030 1038 1059 rBV 14227 37832 15.14% 2.670%

28 13.307 1081 1086 1100 rVB2 5264 11502 4.60% 0.812%

29 13.959 1197 1200 1207 rVV2 3986 9198 3.68% 0.649%

30 14.033 1208 1213 1221 rVB 3389 8062 3.23% 0.569%

Sum of corrected areas: 1416790
