# Supplementary material for: Co-exposure risks of pesticides residues and bacterial contamination in fresh fruits and vegetables under smallholder horticultural production systems in Tanzania
Source: PLoS One. 2020 Jul 15;15(7):e0235345. doi: 10.1371/journal.pone.0235345 (PMC7363064; doi:10.1371/journal.pone.0235345)
Supplement: Attachment — Submitted filename: Comments.docx [file pone.0235345.s004.docx]

**Comments:**

The manuscript entitled “**Co-exposure risks of pesticides residues and bacterial contamination in fresh fruits and vegetables under smallholder horticultural production systems in Tanzania”** is well written and innovative in its aims to investigate the effect of simultaneous exposure of pesticide residues and bacterial contaminants in food matrices. The aim constraint with the manuscript being accepted for publication lies on the fact that the authors have investigated large number of samples set for multiple group of pesticides as well as bacterial contaminants which seems to be statistically relevant. However, there are few queries and comments.

Please increase the resolution of each figures and some of tables can be merged giving better explanation of the co-exposure of the pesticides and bacterial contaminants.

The manuscript needs major revision in terms of the comments provided below.

1. **Abstract**

- From Page 1-3, The whole content in the abstract has been written in duplex. Please correct it.
- Page 1, Line “Vegetables from farms (60.7%) contained more dual contaminants than market-based vegetables” …Any logical explanation behind this?
- Page 2, Line “Immediate actions are therefore required to mitigate the health effects of these toxic and bacterial contaminants” …Specify the action to be taken.

2. **Introduction**

- Page 3, Line “However, one of the health threats is unacceptable pesticide residue levels and bacterial contamination” …. Reframe this sentence
- Page 4, Line “Exposure to pesticides is, therefore, more likely to affect the general population through consumption of contaminated food with pesticide residues (Neff et al., 2012) …. Please use same reference citation pattern as done in whole manuscript.
- Page 4, Line, “as pesticides use had been linked to support and increase microbial growth in vegetable produce” Rewrite the sentence.
- Page 4, Line “However, there is limited information on the co-exposure risks from pesticide residues and pathologic bacterial contaminants in smallholder horticultural production systems in Tanzania” …. Please highlight briefly in the introduction section about the earlier report of co-exposure of pesticides and bacterial contaminants.

3. **Materials and Methods**

- Page 5, section: samples…...What criteria were followed for sample selection and why only 250 samples out of 613 were selected for bacterial contaminants study.
- Please add a couple of section in material and method section such as chemicals and reagent stating the source of the chemicals/reagents purchased. Also add section regarding the standard preparation for pesticides residues.
- Change the title of section “Sample analysis”
- “Recovery, quantitative evaluation and detection limits” Move this section to Results and discussion and elaborate in detail.
- Mention a section for the tools used for the statistical and data analysis

4. Results

- Change the title “Horticultural samples collected”
- Change the title “Nature of pesticides used in vegetable production**”** to class of pesticides…
- This whole section needs language correction

5. Discussion

- This section can be shorten excluding the repeated lines already discussed in the introduction section.
