# Supplementary material for: Co-exposure risks of pesticides residues and bacterial contamination in fresh fruits and vegetables under smallholder horticultural production systems in Tanzania
Source: PLoS One. 2020 Jul 15;15(7):e0235345. doi: 10.1371/journal.pone.0235345 (PMC7363064; doi:10.1371/journal.pone.0235345)
Supplement: Attachment — Submitted filename: Reviewers Comment.docx [file pone.0235345.s005.docx]

Comments on the manuscript entitle **“Co-exposure risks of pesticides residues and bacterial contamination in fresh fruits and vegetables under smallholder horticultural production systems in Tanzani”**

**Comments**

1. The abstract needs restructured as the whole abstract repeated again from line no. 20 “This study was carried out to assess co-exposure…………………”.
2. A detailed method of sample preparation is needed. Usually people consume the vegetable after through washing then why you selected unwashed sample?
3. The procedure of quantitative evaluation of pesticide residue is not clear and it needs explanation with the concentration used.
4. In the result section, the presentation of data is in the very poor form, it need extensive revision and restructure. Most of the data represented in percentage only, the statistical tool needs to apply for the significance of study.
5. Studies have also been reported that microbial fauna in soil become adversely affected through the use of pesticide. How would you correlate it with your present study?
6. The discussion section needs revision to incorporate the other relevant studies and their association with your results.
7. Do you have measured the levels of pesticide residues in the blood and urine of the exposed person? If a person consume the vegetable after washing it properly or by other treatment then what will the effect on pesticide residues and bacterial contamination?
8. The recent references including the pesticide induced adverse health effects in agriculture workers such as “Kori et al 2019 and 2020, Journal of Biochemical and Molecular Toxicology” and others can be included in the manuscript for better making the discussion better.
