# Supplementary material for: Co-exposure risks of pesticides residues and bacterial contamination in fresh fruits and vegetables under smallholder horticultural production systems in Tanzania
Source: PLoS One. 2020 Jul 15;15(7):e0235345. doi: 10.1371/journal.pone.0235345 (PMC7363064; doi:10.1371/journal.pone.0235345)
Supplement: Attachment — Submitted filename: RESPONSES TO REVIEWERS COMMENTS _PLOS_ONE.doc [file pone.0235345.s006.doc]

RESPONSES TO COMMENTS

| **SN** | **SECTION** | **COMMENT** | **RESPONSE** | **ACTION TAKEN** |
| --- | --- | --- | --- | --- |
|  |  | **Reviewer 1** |  |  |
| 1 | General | Please increase the resolution of each figure and some of tables can be merged giving better explanation of the co-exposure of the pesticides and bacterial contaminants. | Figures corrected | Resolution of figured and been increased and some tables merged |
| 2 | **Abstract** | From Page 1-3, The whole content in the abstract has been written in duplex. Please correct it. | Abstract corrected | Duplicate removed |
| 3 |  | Page 1, Line “Vegetables from farms (60.7%) contained more dual contaminants than market-based vegetables” …Any logical explanation behind this? | Logical explanation given | This may have resulted from excessive pesticides use and unhygienic handling of fresh fruits and vegetables at production level. |
| 4 |  | Page 2, Line “Immediate actions are therefore required to mitigate the health effects of these toxic and bacterial contaminants” …Specify the action to be taken. | Action to be taken specified | There is urgent need to develop pesticides monitoring and surveillance systems at farmer level, educating farmers and promoting the use of greener pesticides to mitigate the health effects of pesticides and bacterial contaminants. |
| 5 | **Introduction** | Page 3, Line “However, one of the health threats is unacceptable pesticide residue levels and bacterial contamination” …. Reframe this sentence | The sentence reframed | Unacceptable pesticide residue levels and bacterial contamination of these vegetables resulting from poor agricultural practices, food handling and unhygienic factor increases dietary exposure risks |
| 6 |  | Page 4, Line “Exposure to pesticides is, therefore, more likely to affect the general population through consumption of contaminated food with pesticide residues (Neff et al., 2012) …. Please use same reference citation pattern as done in whole manuscript. | Reference corrected | The same reference citation pattern as done in whole manuscript. |
| 7 |  | Page 4, Line, “as pesticides use had been linked to support and increase microbial growth in vegetable produce” Rewrite the sentence. | Sentence had been rewritten | This is because pesticides use had been linked to support and increase microbial growth in vegetable produce. |
| 8 |  | Page 4, Line “However, there is limited information on the co-exposure risks from pesticide residues and pathologic bacterial contaminants in smallholder horticultural production systems in Tanzania” …. Please highlight briefly in the introduction section about the earlier report of co-exposure of pesticides and bacterial contaminants. | Brief section about the earlier reports of co-exposure of pesticides and bacterial contaminants included in the introduction | Despite this fact, few studies on the effects of co-contamination of fresh vegetables and the resultant health risks had been reported . Paradoxically, no such studies had been reported in Tanzania, while available data elsewhere did not provide comparative levels of simultaneous occurrence and proportions of pesticides and bacterial contaminants in vegetable samples. |
| 9 | **Materials and Methods** | Page 5, section: samples…...What criteria were followed for sample selection and why only 250 samples out of 613 were selected for bacterial contaminants study. | Criteria for sample selection and reasons for using a subsample of 250 bacterial contamination assessment explained | A subsample of 250 was analyzed for bacterial contamination due to homogeneity of samples. Likewise, only samples collected from areas where samples could reach the lab within 4 hours of samples collection were considered for assessment of bacterial contamination. Inclusion criteria were based on the most consumed vegetables. Samples were taken in sterile polyethylene bags which were placed in iced packed cool boxes and transported to the laboratory. Samples were selected according to FAO recommended guideline on sampling for pesticide residue analysis, method of sampling for the determination of pesticides residues where, each whole fruit, vegetable or bunch of vegetables were taken to form a unit, except where these were very small. The minimum weight for small and medium size sample was 1kg and that for large size product was 2 kg. |
| **10** |  | Please add a couple of section in material and method section such as chemicals and reagent stating the source of the chemicals/reagents purchased. Also add section regarding the standard preparation for pesticides residues. | **Section on** chemicals and reagent and standard preparation for pesticides residues included in materials and methods | Chemicals and reagent  Fifty-two (52) certified pesticides standards with purities between 99.0–99.9% were obtained the official registrant companies of specific pesticides in Tanzania. Tetramethrin standard was obtained from Star Import & Export, Tanzania, Pirimiphos–methyl, Permethrin, Profenofos, and Lambda Cyhalothrine from Syngenta Crop Protection Ag, Switzerland; Cabaryl from Bayer Environmental Science, Triadimefon from Meru Agro Tours & Consultant Tanzania, Dimethoate from Sapa Chemical Industrial Ltd, Chlorpyrifos and Bifenthrin from Balton Tanzania Ltd-Tanzania, Oxyfluorfen from Bayer East Africa Ltd-Kenya, Malathion from Tanzania Crop Care Limited, Tanzania while Propanil was obtained Suba Agro-Trading and Engineering Co.Ltd –Tanzania. Solvents at HPLC grade including acetonitrile, acetic acid and acetone, salts of analytical grade such as anhydrous magnesium sulphate, sodium sulphate and sodium acetate; Primary Secondary Amine (PSA), were locally sourced from Immuno Lab Supplies, Dar es Salaam Tanzania.  **Standard preparation for pesticides residues**  Individual pesticide standard stock solutions were prepared in acetone to a final concentration of 20.0 mg/L and stored at -20 0C. The standard mixed component solution was then prepared by diluting each primary standard solution with acetonitrile with 1% acetic acid (1:10 v/v ml). This was then used for spiking extracted fruit and vegetable samples. A 100μl of 1 mg/ml heptachlor was used as an internal standard to ensure accuracy of the GC-MS response. |
| 11 |  | Change the title of section “Sample analysis” | Title section changed | Gas chromatography-mass spectrometry system and operating conditions |
| 12 |  | “Recovery, quantitative evaluation and detection limits” Move this section to Results and discussion and elaborate in detail. | Recovery, quantitative evaluation and detection limits moved to Results and discussion and elaborated in detail. | **Recovery, quantitative evaluation and detection limits**  The method performance for the quantification of concentration of pesticide residues in fresh fruits and vegetables widely produced and locally consumes was validated according to the European Commission guidelines for pesticides analysis . This was done by determining recoveries, Limit of Detection (LOD), Limit of Quantification (LOQ), precision and linearity. Recovery was performed by analyzing mixture of standard pesticides in blank vegetable samples at different known concentrations of 0.1 mg/kg, 0.5mg/kg, 1mg/kg, and 1.5mg/kg in triplicates. A 15 g homogenized sample was spiked with pesticide mixture standard solution and allowed to equilibrate for 3 hours prior to extraction. Extraction and analysis were done according to procedures described previously. Calibration curves constructed from the concentration and peak areas of the chromatograms obtained with standards were used to calculate recovery values. The mean recoveries ranged between 75% and 115%, with an average of 94%. Precision was determined by calculating relative standard deviation (RSD) of the lowest concentration that could show linearity in blank vegetable samples. The relative standard deviations (RSD) obtained was below 10% with an average of 7.7%.  Linearity was determined by analyzing a mixture of pesticide standards at different concentrations ranging from 0.005-0.02mg/kg. The area of the corresponding peak in the sample was then compared with that of the standard. Specificity and validity of the method was monitored by running control blank vegetable samples simultaneously, in which no chromatographic peak was observed at the same retention times of target pesticides which indicated non occurrence of interferences. Analyses were carried out in triplicates and the mean concentrations based on the number of samples that tested positive for each sample calculated. Limits of quantification for the method were calculated by considering a value 10 times that of background noise while detection limits were found by determining the lowest concentrations of the residues in each of the matrices that could be reproducibly measured at the operating conditions of the GC using a signal-to-noise (S/N) ratio of 3. The calculated Limit of detection limits (LOD) ranged from 0.002–0.006 mg/kg while the Limits of Quantification (LOQ) ranged from 0.002 to 0.016. |
| 13 |  | Mention a section for the tools used for the statistical and data analysis | Section on Statistical and data analysis | **Statistical and data analysis**  Statistical analysis of data was done using SPSS 22.0 computer software. Descriptive statistics such as frequencies, percentages, mean, and standard deviations were performed. Chi square (χ2) test was used to determine statistically significant differences in pesticide residues and bacterial contamination rates between vegetables from farms and markets. Pearson correlation test was used to establish the correlation between the levels of pesticide and bacterial contamination. Binary logistic regression analysis was done to determine factors influencing likelihood of simultaneous pesticide and bacterial contamination of vegetables under smallholder vegetable production systems in Tanzania. Significant level for the results was accepted at p < 0.05. |
| 14 | Results | Change the title “Horticultural samples collected” | The title changed accordingly | Pesticides residues in fresh fruits and vegetable samples |
| 15 |  | Change the title “Nature of pesticides used in vegetable production**”** to class of pesticides | The title changed accordingly | Classes of pesticides used and residues detected from fresh vegetable |
| 16 |  | This whole section needs language corrections | Language corrected | English language and grammar had been corrected accordingly. |
| 17 | Discussion | This section can be shorten excluding the repeated lines already discussed in the introduction section. | Discussion section shortened. Repeated lines already discussed in the introduction section excluded | The discussion section had been edited accordingly, repeated lines omitted. |
|  |  |  |  |  |
|  |  | Reviewer 2 |  |  |
| 18 | Abstract | The abstract needs restructured as the whole abstract repeated again from line no. 20 “This study was carried out to assess co-exposure…………………”. | Abstract corrected | Duplicate removed, kindly refer to item 2 above |
| 19 | Materials and methods | A detailed method of sample preparation is needed. Usually people consume the vegetable after through washing then why you selected unwashed sample? | Detailed sample preparation included and reasons explained | Briefly, a whole unwashed 200g sample was homogenized through chopping into small pieces, grinding, and blending. This is because; according to the CODEX Guidelines on Good Practice in Pesticide Residue Analysis, pesticides may tend to collect in the stem area of fruits and on the top of vegetables. Therefore vertical sections must be cut through the stem and centre of fruits and the top and center of vegetables should be chopped and homogenized for analysis . |
| 20 |  | The procedure of quantitative evaluation of pesticide residue is not clear and it needs explanation with the concentration used. | The quantitative evaluation elaborated and concentrations used included in Recovery, quantitative evaluation and detection limits section. | Kindly refer to item 12 above. |
|  | Results | In the result section, the presentation of data is in the very poor form, it need extensive revision and restructure. Most of the data represented in percentage only, the statistical tool needs to apply for the significance of study. | The results section had been extensively revised and restructured. More statistical tools included in the analysis | **Statistical and data analysis**  Statistical analysis of data was done using SPSS 22.0 computer software. Descriptive statistics such as frequencies, percentages, mean, and standard deviations were performed. Chi square (χ2) test was used to determine statistically significant differences in pesticide residues and bacterial contamination rates between vegetables from farms and markets. Pearson correlation test was used to establish the correlation between the levels of pesticide and bacterial contamination. Binary logistic regression analysis was done to determine factors influencing likelihood of simultaneous pesticide and bacterial contamination of vegetables under smallholder vegetable production systems in Tanzania. Significant level for the results was accepted at p < 0.05. |
|  |  | Studies have also been reported that microbial fauna in soil become adversely affected through the use of pesticide. How would you correlate it with your present study? | The possible correlation of the current findings and the adverse effects of pesticide use on microbial fauna in soil had been established.  Key issues on pesticides use and effects on microbial activities in the soils included in the introduction and discussion sections. | **Introduction:** Adverse effect of pesticides use may also affect soil fertility because of the adverse effects of pesticidal chemicals on soil microorganisms . Pesticide sorption can either enhance or decrease microbial degradation rates in soil . Their catabolic gene and respective enzymes are capable of degrading pesticides in highly contaminated areas , indicating a symbiotic association and risks of mutual occurrence of pesticides and microbial contaminants in fresh fruits and vegetables.  **Discussion:** The co-occurrence of pesticides residues and bacterial contaminants from this study indicates the symbiotic existence of pesticides and microbial activities in the environment. Several bacterial and fungal isolates had been reported to have significant ability to carry out the degradation of pesticides. Among many bacterial strained identified to be resistant pesticides are bacterial belonging to genus Enterobacter and Pseudomonas . Likewise, Bacillus thuringiensis had been found to able to degrade malathion while Stenotrophomonas maltophilia and Rhodococcus erythropolis are to be responsible for majority of the degradation of pesticides . Presence of Enterobacter and Pseudomonas bacteria from pesticides contaminated fresh vegetables further suggests the effects of pesticides use on the growth of pathogenic bacteria in the environment.  The results of this study show that the contamination levels of pesticide residues and bacterial contaminants could be perceived as a serious problem as most fresh fruits and vegetables recorded values of pesticide residues far above the MRLs with pathogenic bacteria isolated in higher proportions. Pesticides that exceeded the MRLs was higher in most vegetables that are consumed after raw or semi-cooked such as watermelons, carrots, cucumber, tomatoes, onion and sweet paper. |
|  | Discussion | The discussion section needs revision to incorporate the other relevant studies and their association with your results. | Discussion section had been revised and other relevant studies and their association with our results | Discussion section revised accordingly |
|  |  | Do you have measured the levels of pesticide residues in the blood and urine of the exposed person? If a person consume the vegetable after washing it properly or by other treatment then what will the effect on pesticide residues and bacterial contamination? | Levels of pesticide residues in the blood and urine of the exposed persons were not measured in this study. This is beyond the scope of this study. | Recommendations for further studies on the analysis of levels of pesticide residues in the blood and urine of the exposed person provided |
|  |  | The recent references including the pesticide induced adverse health effects in agriculture workers such as “Kori et al 2019 and 2020, Journal of Biochemical and Molecular Toxicology” and others can be included in the manuscript for better making the discussion better. | Suggested and other relevant references included and discussion improved accordingly | Suggested and other relevant references included and discussion improved accordingly |
